# Exploring symptom-level associations between anxiety and depression across developmental stages of adolescence: a network analysis approach

**DOI:** 10.1186/s12888-023-05449-6

**Published:** 2023-12-13

**Authors:** Yunhan He, Chengrui Wu, Joelle LeMoult, Jiasheng Huang, Yue Zhao, Kaixin Liang, Shiyun Chen, Enna Wang, Liuyue Huang, Xinli Chi

**Affiliations:** 1 School of psychology, Shenzhen University, No. 3688, Nanhai Avenue, Nanshan District, Shenzhen, Guangdong 518060 China; 2 The Shenzhen Humanities & Social Sciences Key Research Bases of the Center for Mental Health, Shenzhen, China; 3 Graduate School of Education, University of Pennsylvania, Philadelphia, PA USA; 4 Department of Applied Social Science, The Hong Kong Polytechnic University, Hong Kong SAR, China; 5 Department of Psychology, University of British Columbia, Vancouver, Canada; 6 Department of Psychology, Sun Yat-sen University, Guangzhou, China; 7 Department of Psychology, Faculty of Social Sciences, University of Macau, Macau, China; 8 School of Psychology, Northwest Normal University, Lanzhou, China

**Keywords:** Adolescence, Anxiety, Depression, Comorbidity, Network analysis

## Abstract

**Background:**

Anxiety and depression often co-occur during adolescence, but the associations between symptoms of these two disorders in this developmental period are not yet fully understood. Network analysis provides a valuable approach to uncover meaningful associations among symptoms and offers insights for prevention and intervention strategies. This study aimed to investigate symptom-level associations between anxiety and depression using network analysis and to identify core symptoms, bridge symptoms, and differences in network structure across different stages of adolescence.

**Methods:**

The cross-sectional study was conducted in March 2022 in Shenzhen, China. Participants completed the Generalized Anxiety Disorder Scale-7 and Patient Health Questionnaire Depression Scale, along with demographic questionnaires assessing age and gender. Chinese adolescents aged 10 to 17 who were in Grades 5 or 6 of elementary school, Grades 1 or 2 of middle school, or Grades 1 or 2 of high school, and who could comprehensively understand and read Chinese were recruited as participants. Students in Grade 3 of middle and high schools were excluded due to their upcoming high school or college entrance examinations. Based on age, participants were categorized into early, middle, and late developmental stages of adolescence.

**Results:**

“Loss of control” was among the most central symptoms in the comorbidity network throughout all three developmental stages; “excessive worry” and “anhedonia” emerged as the core symptoms in early adolescence, and “restlessness” as the core symptom in late adolescence. “Anhedonia,” “sad mood,” and “fatigue” were identified as bridge symptoms between anxiety and depression across all three developmental stages of adolescence. The global strength of the network in middle adolescence was significantly higher compared to the other two stages.

**Conclusion:**

These findings highlight the core and bridge symptoms that require special attention and intervention at each stage of adolescence. Moreover, significantly higher network connectivity in middle adolescence suggests this is a critical period for intervention to prevent the development of comorbid mental disorders.

**Supplementary Information:**

The online version contains supplementary material available at 10.1186/s12888-023-05449-6.

## Background

Adolescence is a period of rapid development and change, as well as a time of frequent mental health problems, especially anxiety and depression, the two most common psychological problems reported during this developmental period [1, 2]. Co-occurrence of the two is referred to as comorbid anxiety and depression (CAD), which can increase treatment difficulty, length of illness duration, worsen prognosis and recovery, and increase rates of disability and suicide compared to the existence of a single disorder [3–5]. According to recent meta-analysis studies by Zhang et al. [6] and Yu et al. [7], the prevalence of anxiety ranges from 26.3 to 27%, while the prevalence of depression ranges from 24 to 28%. Meanwhile, among individuals experiencing anxiety or depression, the prevalence of current or lifetime CAD is reported to range from 10 to 75% [8, 9], indicating that a substantial proportion of adolescents experience both anxiety and depression. Given the adverse consequences and high prevalence of CAD, it is essential to carry out research with the potential to enhance our knowledge of it during this developmental period, thereby promoting public health initiatives in mental health promotion and early prevention.

Both the latent variable model and the network model are used to study CAD [10], offering different perspectives on symptoms and their relationships. The former considers symptoms as mere reflective indicators of underlying diseases and disregards any potential interaction among the symptoms themselves [10, 11]. However, empirical evidence suggests that this theoretical stance does not adequately align with reality [12]. For instance, persistent low mood may result in insomnia, subsequently leading to difficulties in maintaining focus during the day and increased irritability in social activities. Psychopathology network theory provides a more nuanced conceptualization of this issue by shifting the perspective to view symptoms as a causal system. According to this theory, symptoms not only indicate but also contribute to the disorder itself. It is the symptoms, along with their interactions, that effectively constitute the disorder [13, 14]. Thus, comorbidity naturally arises from the interconnected of symptom networks [10]. Moving beyond the mere calculation of total symptom scores to evaluate disease severity, network theory offers a deeper understanding of the importance of individual symptoms in the progression of the disorder.

Indeed, several studies have utilized dynamic symptom interaction networks to explore mental disorders, where symptoms are represented as “nodes” and inter-symptom correlations as “edges” [13]. Some studies in children and adolescents have identified core symptoms and global strength as important characteristics of CAD through network analysis. Core symptoms refer to those that are strongly connected to other symptoms within the network [15]; global strength of a network measures the overall connectivity, with stronger connectivity indicating greater vulnerability and susceptibility to mental disorders [16]. In previous research, McElroy et al. [14] found that “anxious/fearful” and “unhappy/sad” were the core symptoms during the developmental stage of 5–14 years old and observed an increasing trend in the global strength of the network. However, bridge symptoms, which connect symptoms of different disorders [17], were not examined in their study. In another study on CAD conducted by Cai et al. [18] focusing on late adolescence, “sad mood” and “worry too much” were also identified as core symptoms. Additionally, “irritability” and “guilty” were core symptoms as well. The study further identified “guilty,” “sad mood,” and “suicide ideation” as bridge symptoms [18]. However, there is a research gap in fully exploring the comprehensive set of core and bridge symptoms of CAD in the specific developmental stage of adolescence.

Additionally, previous studies have extensively documented the dynamic developmental patterns of anxiety and depression in adolescence, but limited research has specifically focused on understanding the changes that CAD may undergo during this developmental period. For example, McLaughlin and King [19] found that anxiety severity, as measured by the average total score, decreased across adolescence, while depression severity remained stable. Using the tripartite model of CAD, Conway et al. [20] documented that negative affect and anxious arousal dimensions showed a decline over time, whereas the anhedonia dimension did not. McElroy et al. [14] conducted a network analysis study on individuals from ages 5 to 14 and found that symptoms within the CAD network were highly interconnected, and the node strength (an indicator of core symptoms) exhibited developmental variations in this age period.

Therefore, it remains crucial to investigate whether the distinct symptoms of CAD also undergo changes throughout the specific period of adolescence, utilizing network analysis. Network analysis can provide valuable insights by investigating bridge symptoms and core symptoms, as well as evaluating changes in global strength over time. By intervening on bridge and core symptoms to mitigate their mutual influence and prioritizing the allocation of healthcare resources to groups with higher global symptom strength, we can potentially enhance the effectiveness of public health initiatives [17, 21, 22].

Building upon the evolving understanding of the comorbidity of anxiety and depression during adolescence, we hypothesize that the network structure of CAD also differs across developmental stages. Based on previous research among Chinese adolescents [23, 24], the transition from early to late adolescence is marked by changes in school contexts, with significant shifts in academic routines and expectations, school environments, and social groups and relationships. Consistent with this past research, we categorized age into distinct developmental stages: early adolescence (10–12 years), middle adolescence (13–15 years), and late adolescence (16–18 years). The goal of our study is to use network analysis to capture characteristics of CAD and compare symptom-level susceptibility to comorbidity across stages of adolescence. Specifically, we will (1) identify bridge symptoms and core symptoms within the CAD network and (2) compare the network structures across the three stages of adolescence. By accomplishing these objectives, we aim to gain insights into the distinctive features of CAD and examine how its network evolves during different stages of adolescence.

## Methods

### Study design and participants

This study utilized data from a large-scale sampling survey conducted in March 2021 in Shenzhen, one of China’s economically developed cities. The survey, carried out in cooperation with the Educational Science Research Institute of Shenzhen, targeted students aged 10 to 17 in local public elementary, middle, and high schools across all districts of Shenzhen. Inclusion criteria included: (1) students enrolled in Grade 5 or 6 of elementary school or Grade 1 or 2 of middle school or high school; and (2) students with proficiency in Chinese reading and comprehension. Exclusion criteria included students in Grade 3 in middle and high school due to their upcoming high school or college entrance examinations, which may hinder their availability for the survey. Ethical approval was obtained from the Medical Ethics Committee of Shenzhen University, and it conformed to the ethical guidelines of the Helsinki Declaration (approval number: 2,020,005). All participants and their parents were informed about the content and aims of the study, and informed consent was obtained from the legal guardians of all enrolled students.

### Procedure

To facilitate data collection, an online survey questionnaire was imported into the Wenjuanxing platform, a Chinese online survey platform (https://www.wjx.cn/). The survey’s background, purpose, and informed consent were explained on the first page of the questionnaire. The survey was completed online by the students as a class unit in the school computer room during a school day, under the supervision and instruction of a teacher or school staff. The questionnaire took approximately 20 min to complete.

### Measures

Anxiety symptoms were assessed using the Generalized Anxiety Disorder Scale-7 (GAD-7), an effective screening instrument for anxiety symptoms [25]. The scale assesses seven typical symptoms of anxiety disorders using a 4-point scale (none = 0; a few days = 1; more than half time = 2; almost every day = 3), with higher scores representing greater severity of anxiety symptoms in the past two weeks. Total scores can range from 0 to 21 [25]. The Chinese version of the GAD-7 has demonstrated good reliability and validity in Chinese adolescents [26].

Depressive symptoms were assessed using the Patient Health Questionnaire Depression Scale (PHQ-9), which is directly based on the nine DSM-IV diagnostic criteria for major depressive disorder and is particularly well-validated for use in primary care settings [27]. Each item corresponds to a symptom of depression, rated on a 4-point scale (none = 0; a few days = 1; more than half time = 2; almost every day = 3). Higher scores on the PHQ-9 indicate greater severity of depressive symptoms experienced in the past two weeks, with scores ranging from 0 to 27 [27]. The Chinese version of the PHQ-9 has demonstrated good reliability in the Chinese adolescent population [28].

### Statistical analyses

We employed jamovi 2.3.26 for descriptive statistics and two-tailed independent t-tests [29], and R 4.0.2 for constructing network structures for CAD in adolescence [30], including network estimation, network stability, and network differences [31]. A threshold score of 10 or higher on both the GAD-7 and PHQ-9 is used to identify comorbidity [25, 31]. The network analysis followed the standard guidelines for network analysis, which included network estimation and visualization, centrality estimation, network stability, and accuracy estimation [32]. Additionally, we conducted a comparison of network structures based on the approach outlined by Van Borkulo et al. [33].

### Network estimation and visualization

All models were visualized as network graphs, with “nodes” representing items from anxiety and depression scales, and “edges” representing the conditional dependence relationship (i.e., regularized partial correlations) between the “nodes” [34]. The R packages “bootnet” and “qgraph” were used to estimate and construct visual network graphs [32].

Due to the positively skewed distribution of the data, we employed a transformation to convert the data into binary format, based on the criterion of “absence or presence of symptoms.” Scores of “0” were coded as the absence of symptoms and scores “1”, “2”, and “3” were coded as the presence of symptoms. The Ising model was then used to estimate the network structure for the binary data [33]. This model encompasses two sets of parameters: threshold parameters, which indicate whether a variable is endorsed (assigned a value of 1) or not (assigned a value of 0), and pairwise association parameters, which represent the strength of the statistical associations (logistic regression coefficients) between the variables [35]. However, estimating a large number of pairwise association parameters can potentially introduce false-positive edges into the network that does not actually exist [34]. To address this issue, we employed the eLASSO procedure in the IsingFit R-package, as outlined in Epskamp et al.’s 2018 tutorial [32], which incorporates a penalty approach based on the Extended Bayesian Information Criterion (EBIC) with a gamma (γ) value of 0.25. This approach retains only the most significant associations between variables in the network while controlling for false-positive edges.

#### Network centrality

Symptoms that exhibit strong associations with other symptoms within a disorder are identified as core symptoms, while symptoms that are connected to two psychiatric disorders are referred to as bridge symptoms [36–38]. The centrality of nodes is used to determine whether the symptoms can be categorized as core symptoms. We used the R package “qgraph” to compute the centrality indices of nodes within the network [32]. Additionally, the R package “networkTools” was utilized to compute nodes’ bridge centrality to identify bridge symptoms [37]. The three most common used centrality statistics are strength, closeness, and betweenness [17], and the three most common bridge statistics are bridge strength, bridge betweenness, and bridge closeness [37]. However, betweenness and closeness are unstable and indirect and therefore not suitable in the context of symptom networks for mental disorders [38]. As a result, strength and bridge strength were selected as the nodal attributes to be assessed in the current study. Strength represents the sum of the absolute edge weights of a node, representing how well the node is directly connected to other notes [32]. The nodes with node strengths significantly higher than those of other nodes are identified as core symptoms [25]. Bridge strength is defined as the sum of all edges that connect a node to symptoms of another disorder, indicating the node’s total connectivity with other disorders [17, 38]. Symptoms corresponding to nodes with bridge strengths in the top 20% are identified as bridge symptoms [17].

#### Network accuracy and stability estimation

We conducted post-hoc stability analyses to assess the stability and accuracy of both edge weights and centrality parameters by using the bootnet R-package [32]. Firstly, we evaluated the accuracy of edge weights by computing their 95% confidence intervals (CIs) through nonparametric bootstrapping (bootstrap samples = 1000). Secondly, following the approach described by Epskamp et al. [32], we assessed the stability of node centrality by calculating the correlation stability (CS) coefficient using a case-dropping subset (bootstrapped samples = 1000). This involved comparing the correlation of centrality indices (such as strength) between the original sample and a randomly selected subset that excluded 70% of the data. To be considered stable, a centrality index should not significantly differ in the subset sample. Stability was measured using CS coefficients, with a recommended threshold of 0.5, but no less than 0.25 [32]. Thirdly, we utilized the non-parametric bootstrap method to examine whether there were significant differences between each pair of centrality estimations for edges or nodes (bootstrapped samples = 1000, α = 0.05).

#### Network comparison

Ising network models estimated for three adolescence stages were compared using the “Network Comparison Test” (NCT) R package [33]. The comparison was performed with 1000 iterations of a permutation test. NCT assessed differences between two networks in two aspects: (i) global strength, which represents the summed edge-weights of the networks, and (ii) structural invariance, which examines statistically significant changes in relations between variables and nodes. Considering that this study mainly focuses on exploring stage susceptibility, only global strength differences were calculated. Global differences in networks were measured by the Global Strength between two networks, which is the weighted absolute sum of all edges in the network [15, 35].

## Results

### Sample characteristics

A total of 78,428 questionnaires were collected from 135 schools. After excluding invalid questionnaires that were not submitted within the allotted time, contained unidentifiable information, or had excessive repetitive responses, the data from 68,425 valid questionnaires was considered suitable for inclusion in the analyses. Among the 68,425 participants, there were 30,160 individuals in early adolescence (11.47 ± 0.64 years old; male percentage: 53.11%), 28,841 in middle adolescence (13.60 ± 0.72 years old; male percentage: 52.00%), and 9,424 in late adolescence (16.32 ± 0.47 years old; male percentage: 48.12%). The detailed number of individuals by age can be found in Table 1.

**Table 1:** Participant characteristics, overall and stratified by gender, age and developmental stage

Development Stage	Age	Total		Males		Females	
		*n*	%	*n*	%	*n*	%
Overall	10 ~ 17 years	68,425	100	35,543	100	32,882	100
Early adolescence	10 years	2459	3.59	1238	3.48	1221	3.71
	11 years	11,131	16.27	5848	16.45	5283	16.07
	12 years	16,570	24.22	8931	25.13	7639	23.23
Middle adolescence	13 years	15,689	22.93	8136	22.89	7553	22.97
	14 years	9042	13.21	4869	13.70	4173	12.69
	15 years	4110	6.01	1985	5.58	2125	6.46
Late adolescence	16 years	6397	9.35	3023	8.51	3374	10.26
	17 years	3027	4.42	1513	4.26	1514	4.60

### Reliability and validity measures

To assess the internal consistency of the measures used in this study, Cronbach’s alpha coefficients were calculated. Confirmatory factor analysis (CFA) was conducted to evaluate the construct validity of the measurement model. As presented in Table 2, the Cronbach’s alpha values ranged from 0.908 to 0.944, indicating good internal consistency for each of the constructs. CFA also confirmed the good construct validity of the scales at each stage of adolescence.

**Table 2:** Scale reliability and validity

		Cronbach’s alpha	*χ2/df*	CFI	TLI	RMSEA	SRMR
GAD-7	early adolescence	0.938	202.232	0.983	0.975	0.082	0.020
	middle adolescence	0.942	239.691	0.980	0.970	0.091	0.022
	late adolescence	0.944	126.372	0.970	0.954	0.115	0.029
PHQ-9	early adolescence	0.909	269.584	0.949	0.932	0.094	0.036
	middle adolescence	0.914	258.555	0.951	0.935	0.094	0.034
	late adolescence	0.908	93.057	0.944	0.925	0.099	0.038

### Descriptive statistics

The mean anxiety scores across all three developmental stages fall within the “Minimal Anxiety” range of symptom levels [25]. Sample means for depression in the early and middle adolescence stages also fall within the “Minimal Depression” range, while the sample mean for depression during the late adolescence stage falls within the “Mild” range of symptom levels [27]. Results for anxiety and depression scores, as well as comorbidity prevalence rates across three developmental stages, are shown in Table 3, and symptom detection ratios for each stage are shown in Appendix Table S1.

**Table 3:** Prevalence and severity of anxiety and depression in three developmental stages

	Early adolescence (N = 30,160)	Middle adolescence (N = 28,841)	Late adolescence (N = 9424)
Anxiety	2.02 ± 2.58	2.77 ± 2.81	4.41 ± 4.76
Depression	3.07 ± 3.04	3.96 ± 3.18	6.63 ± 5.33
Prevalence of comorbid anxiety and depression	6.60%	11.23%	12.32%

In addition, there were significant differences between the anxiety and depression scores at all developmental stages (*t-test, p < 0.001*). There was no significant difference in comorbidity rates between the middle and late adolescence stages (*t-test, p = 0.15*); however, the early adolescence stage had a significantly lower comorbidity rate compared to the other two stages (*t-test, p < 0.001*).

### Network analysis

#### Network estimation and visualization

**Fig. 1:**
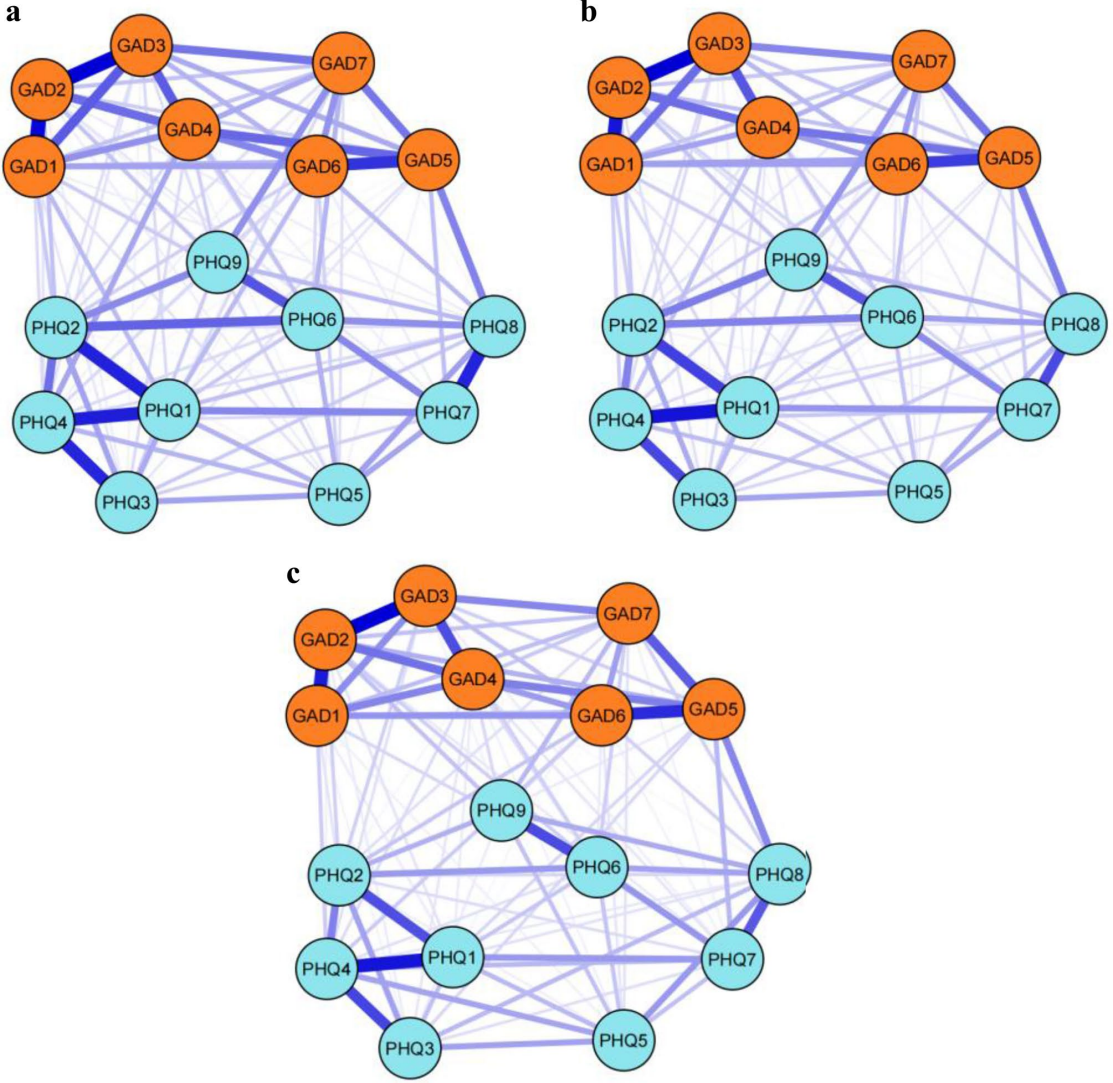
Network of anxiety and depression comorbidity in three developmental stages. (**a**) Network of early adolescence. (**b**) Network of middle adolescence. (**c**) Network of late adolescence. *Notes*: (1) Orange nodes indicate anxiety symptoms; blue nodes indicate depression symptoms. (2) The thickness of each line corresponds to the strength of the correlation. (3) Nodes abbreviation: GAD1: Nervousness; GAD2: Loss of control; GAD3: Excessive worry; GAD4: Trouble relaxing; GAD5: Restlessness; GAD6: Irritability; GAD7: Feeling afraid; PHQ1: Anhedonia; PHQ2: Sad mood; PHQ3: Sleep problems; PHQ4: Fatigue; PHQ5: Appetite changes; PHQ6: Guilt; PHQ7: Difficulty concentrating; PHQ8: Motor problems; PHQ9: Suicidal thoughts

The anxiety and depression comorbidity networks for early, middle, and late adolescence are shown in Fig. 1. The networks illustrate clusters of anxiety and depression symptoms that are interconnected. Among the 120 possible node pairs, 107 node pairs were associated in early adolescence, 106 node pairs in middle adolescence, and 100 node pairs in late adolescence. In all three stages of adolescence, the highest edge weights were between GAD2 (loss of control) and GAD3 (excessive worry) in anxiety symptoms and between PHQ1 (anhedonia) and PHQ4 (fatigue) in depression symptoms.

#### Network centrality

**Fig. 2:**
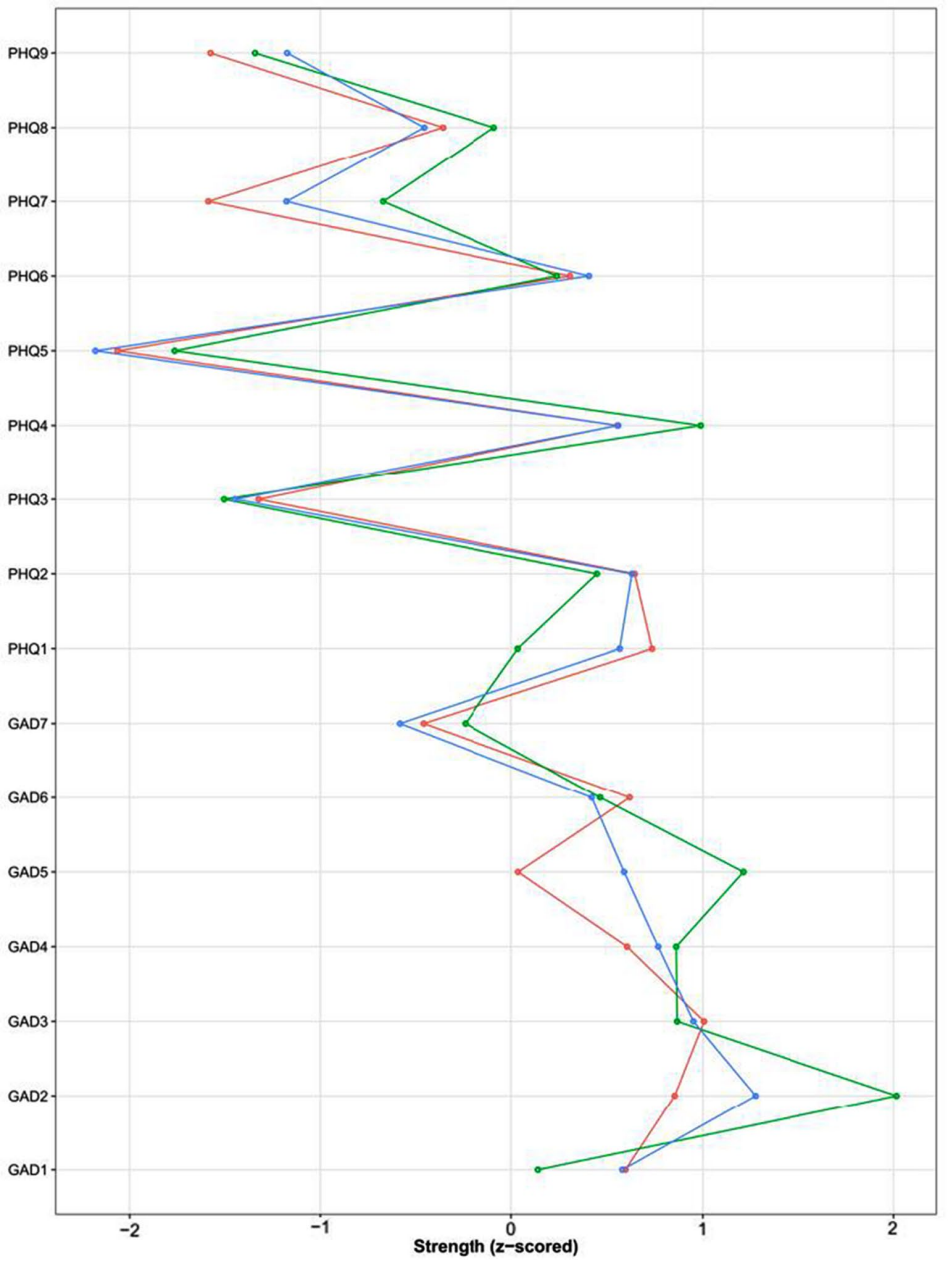
Centrality of anxiety and depression symptoms in three developmental stages. *Note*: (1) Red lines represent early adolescence, blue lines represent middle adolescence, and green lines represent late adolescence. (2) Abbreviation of each node: GAD1: Nervousness; GAD2: Loss of control; GAD3: Excessive worry; GAD4: Trouble relaxing; GAD5: Restlessness; GAD6: Irritability; GAD7: Feeling afraid; PHQ1: Anhedonia; PHQ2: Sad mood; PHQ3: Sleep problems; PHQ4: Fatigue; PHQ5: Appetite changes; PHQ6: Guilt; PHQ7: Difficulty concentrating; PHQ8: Motor problems; PHQ9: Suicidal thoughts

The indices of centrality for symptoms are detailed in Fig. 2, which suggested that GAD2 (loss of control), GAD3 (excessive worry), and PHQ1 (anhedonia) were central (core symptoms) in the early adolescence stage; GAD2 (loss of control) was the core symptom in the middle adolescence stage; GAD2 (loss of control) and GAD5 (restlessness) were the core symptoms in the late adolescence stage. The results of the difference test are depicted in Appendix Figure S1.

**Fig. 3:**
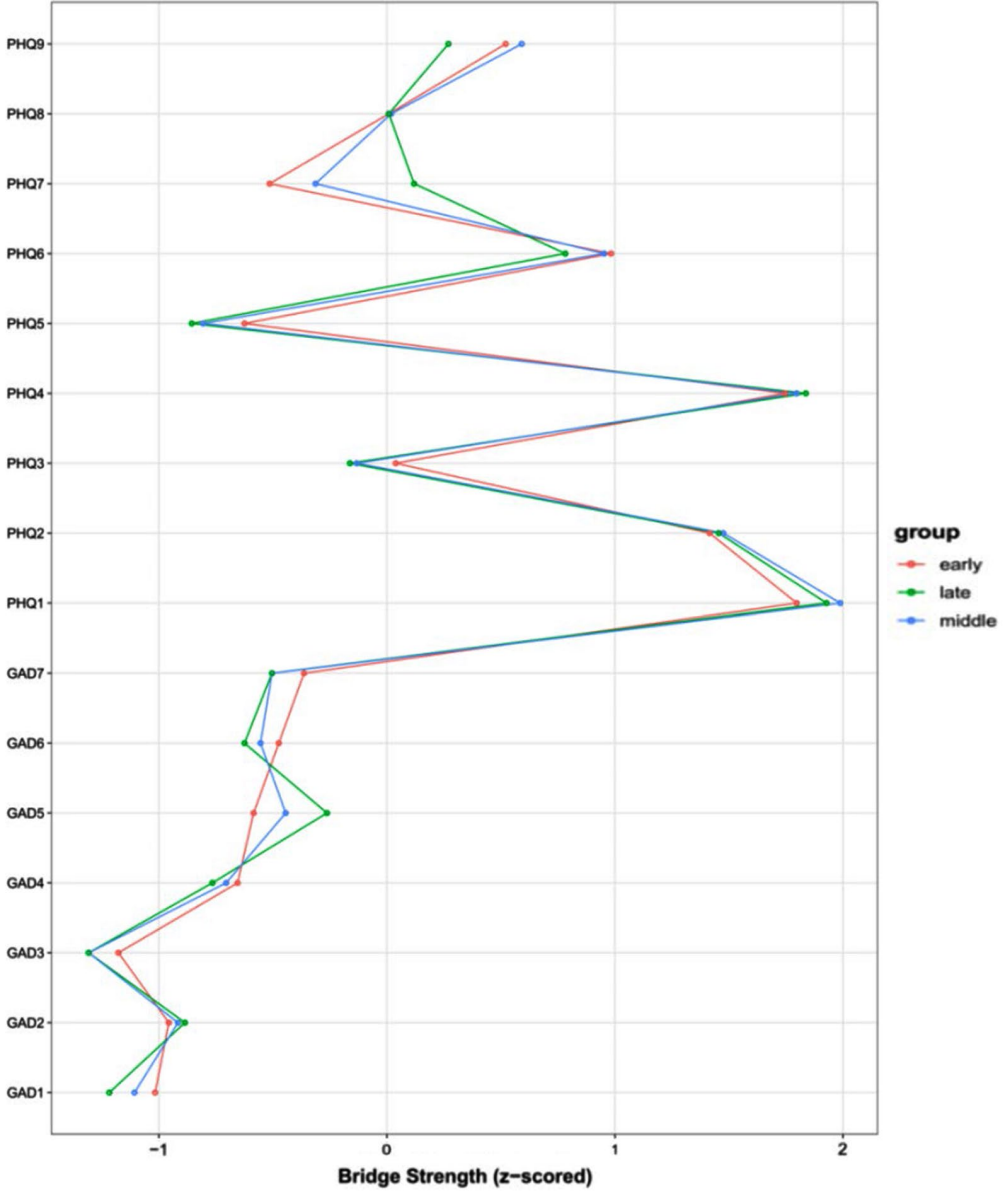
Bridge centrality of anxiety and depression symptoms in three developmental stages. *Note*: (1) Red lines represent early adolescence, blue lines represent middle adolescence, and green lines represent late adolescence. (2) Abbreviation of each node: GAD1: Nervousness; GAD2: Loss of control; GAD3: Excessive worry; GAD4: Trouble relaxing; GAD5: Restlessness; GAD6: Irritability; GAD7: Feeling afraid; PHQ1: Anhedonia; PHQ2: Sad mood; PHQ3: Sleep problems; PHQ4: Fatigue; PHQ5: Appetite changes; PHQ6: Guilt; PHQ7: Difficulty concentrating; PHQ8: Motor problems; PHQ9: Suicidal thoughts

The bridge strengths of the network symptoms are presented in Fig. 3 for the three stages of adolescence. PHQ1 (anhedonia), PHQ2 (sad mood), and PHQ4 (fatigue) appeared to have the highest bridge strengths in the CAD network in all three developmental stages, making them the symptoms that bridge the anxiety and depression networks.

#### Network stability and accuracy

The results of the edge weight bootstrap procedure are presented in Fig. S2, which demonstrates that the edge weights in the current sample are consistent with those in the bootstrap sample, indicating a high stability of the network. Figure  S2 displays the findings of the Subsetting Bootstrap analysis, revealing that the stability coefficients (CS-Coefficient) for node strength were consistently high across all three stages of adolescence, with a value of 0.75. Additionally, the CS-Coefficient for node bridge strength was above the recommended threshold of 0.5 in all three stages, with values of 0.67, 0.59, and 0.52. These results suggest that both the nodes and edges of the network exhibit high stability and accuracy.

#### Network comparison test

The global strengths of the three networks were 47.68, 48.14, and 48.91, with significant differences between the early and middle stages, and the early and late stages, and no significant differences between the middle and late stages (early vs. middle: *diff* = 0.46, *p* = 0.04; early vs. late: *diff* = 1.23, *p* = 0.03; middle vs. late: *diff* = 0.77, *p* = 0.15).

## Discussion

In the current study, we conducted a network analysis to explore the network structures of CAD and susceptibility differences to CAD in early, middle, and late adolescence. We identified several core symptoms of CAD, as well as bridge symptoms between anxiety and depression in the CAD network. First, “loss of control” emerged as the core CAD symptom throughout all three developmental stages. “Excessive worry” and “anhedonia” were identified as core symptoms in early adolescence, and “restlessness” was a core symptom in late adolescence. Second, when concentrating on the overlap between anxiety and depression, “anhedonia,” “sad mood,” and “fatigue” were the bridge symptoms across all three developmental stages, suggesting that these symptoms connect and transmit comorbidity between anxiety and depression. In examining the network structures across three developmental stages, the data revealed that the middle adolescence stage exhibited the highest global strength, with a significant difference observed when comparing the middle to the early adolescence stages. Although there was no statistically significant difference between the middle and late adolescence stages, the middle stage’s global strength remained the highest. This suggests that the middle stage of adolescence may be a period of increased vulnerability and susceptibility to mental disorders, including CAD.

### CAD core symptoms

“Loss of control” was identified as highly central in the CAD network throughout the three adolescent stages. This finding aligns with previous studies conducted in different populations. For instance, in studies involving patients diagnosed with both anxiety and depression in Germany (42.24 ± 14.38 years old, *n* = 5614) [39], and patients with epilepsy in China (31.5 ± 2.3 years old, *n* = 313) [40], “loss of control” was also identified as a core symptom. Despite the differences in demographics, associated life stressors, and diagnostic status of participants, the consistency in identifying “loss of control” as a core symptom suggests its significance in anxiety-depression comorbidity.

The presence of “loss of control” as a core symptom across diverse populations indicates its potential as a hallmark symptom in the development of CAD. Psychoeducation and cognitive-behavioral therapy (CBT) have shown effectiveness in improving emotion regulation skills, which can help individuals better cope with feelings of losing control. For example, meta-cognitive therapy can be used to modify adolescents’ beliefs about worry, shifting the focus from the concern itself to their thoughts about the concern [41]. By targeting “loss of control” as a core symptom and incorporating interventions such as psychoeducation and CBT, it may be possible to enhance emotion regulation and reduce the severity of anxiety and depression comorbidity. This highlights the importance of implementing targeted interventions that address specific core symptoms to improve overall mental well-being in adolescents.

Additionally, in early adolescence, “excessive worry” and “anhedonia” were two core symptoms specific to this stage. Early adolescence typically encompasses the age range of 10–12 years. During this period, self-awareness is gradually enhanced, and adolescents actively explore the external world while facing new tasks and challenges independently [42]. It is not uncommon for them to encounter failure and experience heightened frustration as they navigate these new experiences. Prolonged experiences of failure can lead to a diminished interest in various activities, which is captured by the symptom of “anhedonia” [43]. Given the unique challenges faced by early adolescents, it is crucial to implement preventative measures and targeted interventions for these two core symptoms. One approach could involve equipping early adolescents with coping skills and providing them with resources to effectively navigate failure and manage excessive worry. By empowering early adolescents with appropriate strategies, they can develop resilience and adaptive responses to challenging situations, potentially mitigating the risk of anxiety and depression comorbidity.

On the other hand, “restlessness” was another core symptom in late adolescence, which also requires targeted preventative measures and interventions. Interestingly, this symptom has also been identified as a core symptom of CAD in Chinese adults following the peak of the pandemic [44]. Stressful events and circumstances have been found to play a significant role in the manifestation of restlessness [45].

In late adolescence, individuals aged 16–18 in China face a particularly stressful event: a college entrance examination, which is an important event for students and their families. During this final stage of adolescence, individuals are not only burdened by the weight of academic expectations and uncertainties about their future educational and career paths, but also face pressure from teachers and parents [46, 47]. These demanding and stressful circumstances can contribute to a state of restlessness among adolescents.

To relieve or alleviate this core symptom, it is essential to provide effective stress management techniques and coping strategies that can help adolescents manage their stress levels. Approaches such as self-compassion, mindfulness, regular exercise, and relaxation techniques like listening to music can be valuable tools in reducing restlessness and promoting emotional well-being [48–51]. Furthermore, it is important for parents and educators to support late adolescents during this critical period of transition into adulthood by providing resources, support systems, and fostering open communication. By creating a nurturing and understanding environment, adolescents can better cope with the stressors they face and build resilience as they navigate this challenging phase of their lives.

### CAD bridge symptoms

We found that “anhedonia,” “sad mood,” and “fatigue” may bridge the associations between anxiety and depression and contribute to the development and maintenance of CAD. Similar bridge symptoms have been reported in studies involving different populations, such as migrant Filipino domestic workers in Macao and Chinese nursing students and adolescents [18, 52–54]. It has been proposed that anxiety may transform into depression through the manifestation of “anhedonia” [55]. Anhedonia is associated with a decrease in desire, interest, and reinforcement learning [56, 57]. Individuals experiencing prolonged stress and anxiety may progress to a depleted phase characterized by symptoms of fatigue, sad mood, and loss of motivation [58], thereby triggering a depressed state. Additionally, the tripartite model suggests that negative affectivity, such as “sad mood,” is common to both anxiety and depression [59]. The sustained arousal caused by worry may lead to emotional and physical exhaustion, eventually resulting in low mood and the development of other depressive symptoms [32]. In this case, the symptom of “sad mood” may reflect a progression from anxiety to depression. These findings contribute to our understanding of CAD in adolescence and highlight potential avenues for intervention and prevention strategies aimed at targeting specific core symptoms and bridge symptoms associated with CAD.

Furthermore, the bridge symptom of “fatigue” may be linked to the somatization of psychological distress, which is particularly prevalent in Asian populations, including China [60]. Cultural norms that prioritize the suppression of personal emotions and the persistent stigma surrounding mental health problems contribute to this phenomenon [61]. Adolescents, in particular, are more likely to somaticize their mental health issues rather than directly addressing them [39]. This tendency is further corroborated by qualitative research that examines the stress and coping strategies of adolescents in a Chinese population, where avoidance of direct confrontation with mental health issues is common, and somatization can be a culturally embedded response to distress [62–64].

Besides, Chinese adolescents face excessive burdens that may contribute to their fatigue [40]. They are often required to dedicate extensive time to academic pursuits, leaving little time for entertainment, social activities, and even adequate sleep. The cumulative effect of these demands on their physical and mental well-being can manifest as fatigue. Overall, the cultural context and the high levels of stress and responsibilities experienced by Chinese adolescents contribute to the prominence of “fatigue” as a bridge symptom in CAD. Recognizing these cultural and contextual factors is crucial for understanding the unique manifestations of CAD in this population and for developing targeted interventions and support systems.

### Global strength differences in three developmental stages

The global strength of the network during middle adolescence was found to be higher compared to early and late adolescence, with a significant difference observed between early and middle adolescence. This finding aligns with a previous study by Cai et al. [18], which also reported significant differences in network global strength between junior and senior secondary school grades. Another study by McElroy et al. [65] observed a higher connectivity in anxiety-depression comorbidity symptom networks at age 14 compared to ages 5 and 6. Despite variations in age and region, these studies collectively suggested that symptoms evolve and may reinforce each other during development, with middle adolescence emerging as a critical period for rapid comorbidity development. The heightened network connectivity observed in this stage suggests that middle adolescence may be a particularly vulnerable time for the onset of mental disorders.

### Potential implications

These findings have important potential clinical implications. First, these results highlight the identified core and bridge symptoms as crucial in the development and maintenance of CAD, implying shared mechanisms through which the symptomatology is associated with anxiety and depression. Paying attention to these symptoms is vital for developing interventions that can broadly reduce the risk for CAD in adolescents. Furthermore, our study highlights the severity of CAD in adolescents and demonstrates a progressive increase in susceptibility to the development and persistence of CAD. Specifically, the transition from early to middle adolescence appears to be a critical period for the emergence of CAD, suggesting this specific age range as a target for intervention.

### Limitations and future research

There are several limitations that should be acknowledged. Firstly, although network theory implies a causal account of the evolution of psychiatric syndromes [36], the cross-sectional nature of our data prevents us from revealing causal dynamics, especially in relation to the direction of relationships between anxiety and depression symptoms [5, 66]. As mentioned before, future longitudinal studies are needed to explore the activation of symptoms over time and provide insights into the causal relationships between symptoms [67].

Secondly, our study only included DSM-5 symptoms of anxiety and depression, while non-DSM-5 symptoms may also play a significant role in symptom networks [68]. Hence, including a more comprehensive set of symptoms and scales in future studies would provide a more nuanced understanding of the networks. Besides, it is important to acknowledge that symptoms from other disorders, such as post-traumatic stress disorder and eating disorders [69, 70], may co-occur with anxiety and depression. Future research should consider incorporating these common adolescent mental health problems and their symptoms into network analyses to identify pivotal symptoms for prevention and intervention efforts.

Furthermore, the sample used in this study consisted of adolescents from a school setting, which may limit the generalizability of the findings to the broader community or clinical populations. Variations in network connectivity across different samples or populations have been observed in prior research. Therefore, caution should be exercised when extending the results to clinical settings, and it would be valuable to include clinical samples in future investigations to determine the consistency or discrepancies in the conclusions.

Lastly, considering cultural differences, it is important to note that the manifestation of emotions as somatic symptoms may more normally present in collectivist societies [71]. While our study focused on a specific cultural context, further research is needed to verify the results in non-collectivist countries and explore the influence of cultural factors on symptom networks.

## Conclusion

In summary, the current study characterized the comorbidity of anxiety and depression network within a large Chinese adolescent sample by employing network analysis. Through the examination of network structures, we identified core symptoms (e.g., “loss of control,” “excessive worry,” “anhedonia,” and “restlessness”) and bridge symptoms (e.g., “anhedonia,” “sad mood,” and “fatigue”) that were consistent across all three stages of adolescence. We also identified middle adolescence as the critical period for the rapid development of CAD.

Overall, these findings provide valuable insights into the understanding of anxiety-depression comorbidity in adolescents and emphasize the need for targeted interventions and preventive measures during specific stages of adolescence. Future work is needed to incorporate these core and bridge symptoms into the design and development of early detection and intervention strategies to address the unique challenges faced by adolescents in managing anxiety and depression.

### Electronic supplementary material

Below is the link to the electronic supplementary material.

**Supplementary Material 1:** Supplementary Tables and Figures

## Data Availability

The datasets used and/or analyzed during the current study are available from the corresponding author on reasonable request.

## Abbreviations

CAD: Comorbid Anxiety and Depression
GAD: Generalized Anxiety Disorder Scale
PHQ: Patient Health Questionnaire Depression Scale
CFA: Confirmatory factor analysis
EBIC: Extended Bayesian Information Criterion
CS: Correlation Stability
NCT: Network Comparison Test
CBT: Psychoeducation and cognitive-behavioral therapy
