# Supplementary material for: Exploring symptom-level associations between anxiety and depression across developmental stages of adolescence: a network analysis approach
Source: BMC Psychiatry. 2023 Dec 13;23:941. doi: 10.1186/s12888-023-05449-6 (PMC10720222; doi:10.1186/s12888-023-05449-6)
Supplement: Supplementary file 1 — Supplementary Material 1: Supplementary Tables and Figures [file 12888_2023_5449_MOESM1_ESM.docx]

**Appendix**

Table S1 Symptoms detection ratios for Each Stages

| Symptoms(Abbreviations) | Early Adolescence  (N=30,160） | Middle adolescence  (N=28,841） | Late adolescence(N=9,424） |
| --- | --- | --- | --- |
| GAD1: Nervousness | 31.98% | 45.31% | 71.46% |
| GAD2: Loss of control | 25.99% | 37.53% | 64.75% |
| GAD3: Excessive worry | 30.67% | 43.94% | 74.34% |
| GAD4: Trouble relax | 29.13% | 40.90% | 68.88% |
| GAD5: Restlessness | 22.52% | 30.32% | 46.05% |
| GAD6: Irritability | 32.36% | 43.24% | 65.76% |
| GAD7: Feeling afraid | 28.92% | 35.42% | 49.88% |
| PHQ1: Anhedonia | 49.11% | 61.15% | 91.68% |
| PHQ2: Sad mood | 39.85% | 51.90% | 78.83% |
| PHQ3: Sleep problem | 31.13% | 39.52% | 74.54% |
| PHQ4: Fatigue | 38.30% | 54.22% | 92.42% |
| PHQ5: Appetite | 35.53% | 41.36% | 65.97% |
| PHQ6: Guilty | 36.89% | 49.01% | 79.66% |
| PHQ7: Difficulty concentrating | 28.41% | 38.60% | 68.24% |
| PHQ8: Motor | 25.80% | 32.11% | 48.29% |
| PHQ9: Suicide | 21.96% | 28.03% | 36.45% |

1. early adolescence (b)middle adolescence (c)late adolescence


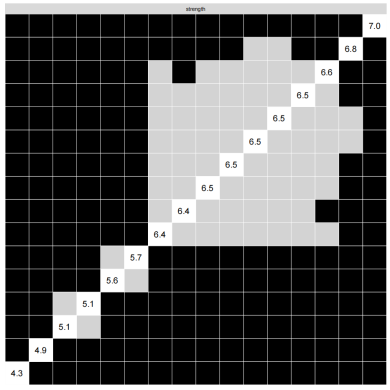

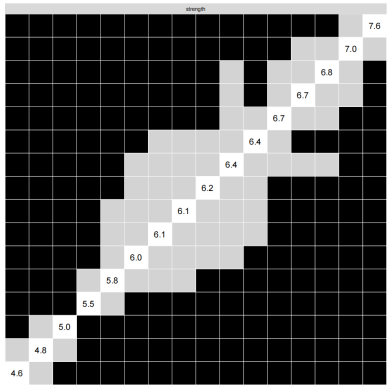

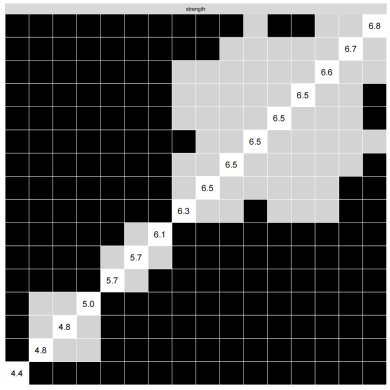


**Figure S1 .** Nonparametric bootstrapped difference test for strength.

Note: Gray boxes indicate no difference between nodes, whereas black boxes indicate significant difference (α = 0.05). Values reported in the diagonal represent the strength values of each node.

1. early adolescence (b)middle adolescence (c)late adolescence


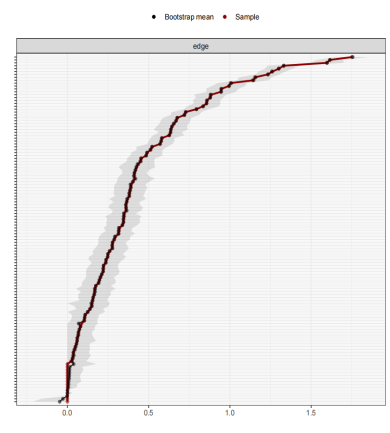

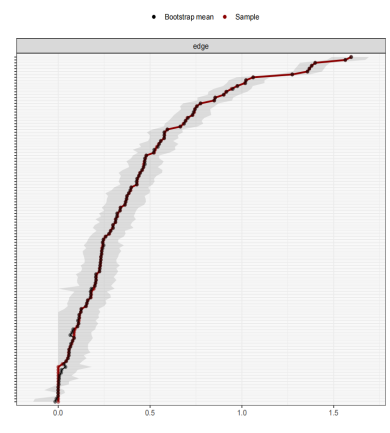

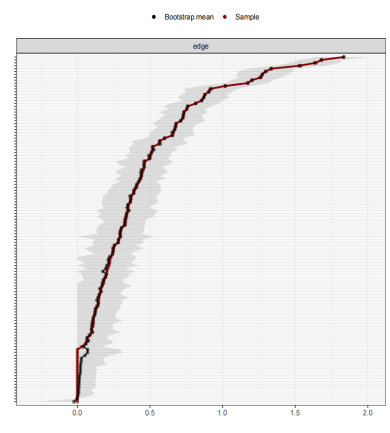


**Figure S2.** Nonparametric bootstrapped difference test for Stability of Edge Weights

Note: The x-axis indicates the edge weights and the y-axis indicates the nodes linked by the edges. The black dots denote the mean value of the bootstrapped edge weights and the red dots denote the edge weights from current sample. The black lines denote the 95% confidence intervals of the bootstrapped sample.

(a)early adolescence (b)middle adolescence (c)late adolescence


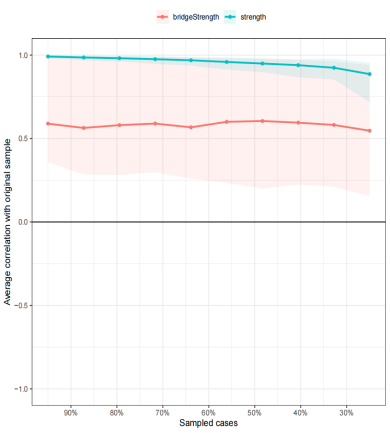

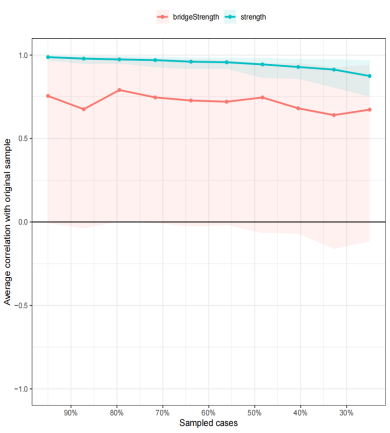

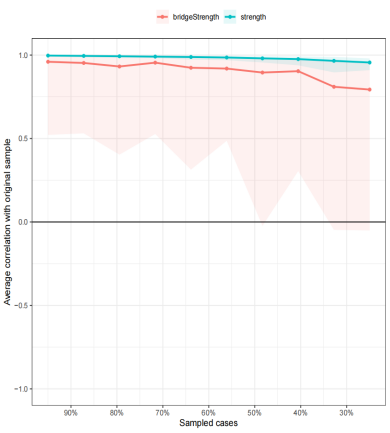


**Figure S3.** Stability of nodes strength and bridge strength

Notes: The x-axis indicates the included portion of cases, and the y-axis indicates the correlations between the original centrality indices with the estimated centrality after dropping part of the cases. Lines with different colors represent different network properties, green color represent strength and red color represent bridge strength. The shades indicate the range from the 2.5th quantile to the 97.5th quantile.
